# A first-in-human phase 1 study of a hepcidin monoclonal antibody, LY2787106, in cancer-associated anemia

**DOI:** 10.1186/s13045-017-0427-x

**Published:** 2017-03-21

**Authors:** Saroj Vadhan-Raj, Rafat Abonour, Jonathan W. Goldman, David A. Smith, Christopher A. Slapak, Robert L. Ilaria, Ramon V. Tiu, Xuejing Wang, Sophie Callies, Joanne Cox, Jay L. Tuttle, Yiu-Keung Lau, Eric J. Roeland

**Affiliations:** 1 UT M.D. Anderson Cancer Center, 1515 Holcombe Blvd, Unit 450, Houston, TX 77030 USA; 2 Indiana University Simon Cancer Center, Indianapolis, IN USA; 3 David Geffen School of Medicine at the University of California, Los Angeles, Santa Monica, CA USA; 4 Compass Oncology, Vancouver, WA USA; 5 Eli Lilly and Company, Indianapolis, IN USA; 6 Eli Lilly and Company, Neuilly-sur-Seine, France; 7 Eli Lilly and Company, Surrey, UK; 8 Moores Cancer Center, University of California, San Diego, La Jolla, CA USA

**Keywords:** Hepcidin, Ferritin, Iron, Cancer, Anemia

## Abstract

**Background:**

Hepcidin plays a central role in iron homeostasis and erythropoiesis. Neutralizing hepcidin with a monoclonal antibody (mAb) may prevent ferroportin internalization, restore iron efflux from cells, and allow transferrin-mediated iron transport to the bone marrow. This multicenter, phase 1 study evaluated the safety, pharmacokinetics (PK), pharmacodynamics (PD), and efficacy of a fully humanized mAb (LY2787106) with high affinity for hepcidin in cancer patients with anemia.

**Methods:**

Thirty-three patients with hepcidin levels ≥5 ng/mL received LY2787106 either every 3 weeks (19 patients, dose range 0.3–10 mg/kg) (part A) or weekly (14 patients, dose 10 mg/kg) (part B). LY2787106 PK/PD markers of iron and hematology biology were measured.

**Results:**

LY2787106 clearance (32 mL/h) and volume of distribution (7.7 L) were independent of dose and time, leading to a dose-proportional increase in concentration with dose. Consistent dose-dependent increases in serum iron, and transferrin saturation were seen at the 3 and 10 mg/kg dose levels, typically peaking within 24 h after LY2787106 administration and returning to baseline by day 8.

**Conclusions:**

Our findings indicate that LY2787106 was well tolerated in cancer patients with anemia and that targeting the hepcidin-ferroportin pathway by neutralizing hepcidin resulted in transient iron mobilization, thus supporting the role of hepcidin in iron regulation.

**Trial registration:**

ClinicalTrial.gov, NCT01340976

**Electronic supplementary material:**

The online version of this article (doi:10.1186/s13045-017-0427-x) contains supplementary material, which is available to authorized users.

## Background

Anemia is common in cancer patients, with a prevalence of 30 to 90% depending upon disease stage and the hemoglobin level used to define anemia [1]. It causes fatigue, dyspnea, and other related symptoms that significantly lower the quality of life and functional capacity of patients with cancer. Furthermore, in one systematic review of published studies, anemia was associated with an increased risk of death in cancer patients by up to 75% [2]. The pathophysiology of anemia in cancer is complex and has been attributed to inflammation that leads to increased levels of cytokines that impair the production of hematopoietic growth factors, including erythropoietin, and the bone marrow response to erythropoietin [3].

A common limiting factor in red blood cell (RBC) production is the macrophage’s failure to release sufficient iron for erythroid progenitor cells. Hepcidin is a 25-amino-acid peptide that binds to ferroportin and causes internalization and degradation of the hepcidin-ferroportin complex, leading to decreased iron absorption and reduced iron export from macrophages [4]. High hepcidin levels are found in cancer patients and implicated in anemia pathogenesis [5–7]. It is hypothesized that neutralizing hepcidin with a monoclonal antibody will prevent internalization of ferroportin, restore iron efflux from macrophages, and allow transferrin-mediated iron transport to the bone marrow to support erythropoiesis.

LY2787106 is a fully humanized monoclonal antibody with high affinity for human hepcidin. In this first-in-human phase 1 study, the safety, pharmacokinetics (PK), pharmacodynamics (PD), and efficacy (effects on serum iron panel, reticulocytes, and hemoglobin) of LY2787106 were evaluated in cancer patients with anemia.

## Methods

### Study design

This was a multicenter phase 1 study in anemic patients with cancer and measurable serum hepcidin levels (assay sensitivity, ≥5 ng/mL). The study had two parts. In part A, the primary objective was to assess the safety of LY2787106 over the dosing range (0.3–10 mg/kg intravenously (IV) every 3 weeks (Q3W)). In part B, the primary objective was to assess mean change in hemoglobin from baseline to end of week 12 following LY2787106 treatment (10 mg/kg IV weekly) with and without oral iron supplementation. Secondary objectives were to characterize the PK of LY2787106 and to characterize PD changes in serum iron panel measures, reticulocyte count, reticulocyte hemoglobin content (CHr), and hemoglobin levels after administration of LY2787106.

In part A, an open-label 3 + 3 dose escalation design was used to evaluate the safety and PK and PD effects of LY2787106. In part B, a modified 3 + 3 safety lead-in design was used to enroll patients to once-weekly dosing with LY2787106 without oral iron supplementation before opening enrollment into two separate cohorts that received the study drug with and without oral iron, respectively. All patients entered follow-up once study drug treatment was discontinued.

This study was conducted in accordance with the International Conference on Harmonisation Guidelines for Good Clinical Practice and the Declaration of Helsinki after approval by each site’s institutional review board. All patients gave written informed consent before undergoing any study-specific procedures. This study was registered at www.clinicaltrials.gov as #NCT01340976.

### Patient population

Eligible patients were ≥18 years old with previously treated metastatic or incurable nonmyeloid cancers, hemoglobin <11 g/dL, serum hepcidin levels ≥5 ng/mL (serum hepcidin levels in normal individuals and patients have been previously described [8]), and Eastern Cooperative Oncology Group (ECOG) performance status ≤2. Treatment with chemotherapy was permitted during the course of the clinical trial. A full list of inclusion and exclusion criteria is available in Additional file 1.

### Treatment

#### Part A (dose escalation)

Patients in part A received LY2787106 IV at a dose of 0.3, 1, 3, or 10 mg/kg over 30 min. The first cycle defined the period that governed dose escalation. The criteria for dose-limiting toxicities (DLTs) included any clinically significant grade ≥2 toxicity or other event deemed significant by the investigator, grade ≥3 anemia (excluding patients with baseline <9.0 g/dL) or hemoglobin decrease >1.0 g/dL if baseline hemoglobin was <9.0 g/dL (confirmed by two independent measurements), grade ≥3 cytokine release syndrome/acute infusion reaction, or other grade ≥3 hematological toxicity. Stopping rules are described in Additional file 1. The maximum tolerated dose (MTD) for LY2787106 was defined as the highest tested dose below the level at which at least one third of patients experienced a DLT.

Patients who had no DLT; met no stopping rules; and had increased serum iron, transferrin saturation (TSAT), reticulocyte count, or hemoglobin levels after the initial dose were allowed to receive up to two additional doses at 3-week intervals at the investigator’s discretion.

#### Part B

Interim analysis of part A results showed that LY2787106 given Q3W was well tolerated, had a short half-life (t_1/2_) (~7 days), and led to transiently increased serum iron levels; therefore, the decision was made to use once-weekly dosing in part B. In addition, part B was revised to enroll an additional cohort of patients who would receive oral iron supplementation to determine the safety of the combination and to explore whether oral iron therapy can augment the effects of LY2787106 on iron-restricted anemia.

Patients in part B received eight weekly doses of 10 mg/kg LY2787106 given IV over 30 min with or without daily oral iron supplementation (65–72 mg elemental iron daily) (cohorts B2 and B1, respectively). Patients who met no stopping rules and were considered to be benefiting during the defined treatment period were allowed to receive up to eight additional weekly doses at the investigator’s discretion. The primary efficacy endpoint was mean change in hemoglobin level from baseline to the end of cycle 4 (i.e., week 12).

### Safety assessments

Adverse events were collected and graded according to National Cancer Institute Common Terminology Criteria for Adverse Events (NCI CTCAE) version 3.0 and, when necessary, the Medical Dictionary for Regulatory Activities (MedDRA) version 17.1. Laboratory monitoring included hematology, chemistry, urinalysis, biomarkers, iron panel, serum hepcidin, immunogenicity, and pregnancy test. Selected laboratory results of interest included serum iron, serum hepcidin, hemoglobin, reticulocyte count, TSAT, interleukin-6 (IL-6), serum erythropoietin, ferritin, CHr, soluble transferrin receptor, tumor necrosis factor-α (TNF-α), C-reactive protein, total iron binding capacity (TIBC), and immunogenicity data.

### Pharmacokinetic assessments

Pharmacokinetic analysis was conducted on serum samples from patients in parts A and B who had received at least one dose of LY2787106 and had sufficient postdose samples collected to allow estimation of PK parameters. Serum LY2787106 concentrations were determined by a validated enzyme-linked immunosorbent assay (ELISA). Maximum concentration (C_max_), area under the concentration-time curve (AUC), half-life (t_1/2_), volume of distribution (V_d_), clearance (CL), and other relevant parameters of LY2787106 were calculated as applicable.

### Pharmacodynamic assessments

Pharmacodynamic changes from baseline after administration of LY2787106 were analyzed for key parameters including serum iron, ferritin, TSAT, reticulocyte count, hemoglobin, and serum hepcidin. Other PD parameters analyzed were IL-6, TNF-α, C-reactive protein, and erythropoietin.

### Statistical analyses

The total sample size for this study was determined by the incidence of DLTs (at least 3 evaluable patients per dose cohort and up to 6 patients per dose group in part A prior to establishing the MTD). In part B, the plan was to treat approximately 32 patients so that each of cohorts B1 and B2 would have at least 12 evaluable patients, assuming an approximately 25% early dropout rate. Assuming an improvement in mean hemoglobin of 0.75 g/dL from baseline to the end of cycle 4 and a standard deviation in hemoglobin change of 1.5 g/dL, a sample size of 12 patients in each cohort in part B was determined to have 80% power to detect a statistically significant increase in hemoglobin with a 1-sided type I error rate of 0.20.

Safety analyses were based on data from all patients who had received at least one dose of LY2787106 (safety population). Efficacy analysis in part B was based on data from patients who had received all four of the first four weekly doses of LY2787106 (evaluable population), were at least 60% compliant with oral iron therapy during the first 4 cycles of LY2787106 (cohort B1 only), and had hemoglobin assessed after each of the first four doses. The primary efficacy analysis (part B only) estimated and compared mean changes in hemoglobin from baseline to the end of cycle 4 with the hypothesized null control using a 1-sided 80% confidence interval (CI) (equivalent to a 2-sided 60% CI), based on a *t* distribution. For cohorts B1 and B2, estimates and 2-sided 90% CIs were calculated for the least-squares mean of hemoglobin changes by the repeated-measures method (linear mixed-effects model with baseline value and time as covariates) at each assessment time point and for the AUC for hemoglobin change during the postbaseline treatment period (week 12); the estimates were then compared between the two cohorts.

Pharmacokinetic parameters were computed by standard noncompartmental analysis methods. Data were also analyzed using nonlinear mixed-effect modeling (as implemented in NONMEM). Data from all patients were pooled for analysis to determine compartmental PK parameters and between- and within-patient variability. The primary parameters analyzed were C_max_ and AUC of LY2787106. Other parameters analyzed were t_1/2_, V_d_, and CL. The primary parameters (C_max_ and AUC) were evaluated statistically to delineate the effects of dose proportionality using methods described previously [9]. Least-square estimates of geometric means and corresponding 90% CIs were determined for each dose, together with the dose-normalized ratio of geometric means and CIs.

The absolute percent change from baseline for all PD and immunogenicity endpoints was summarized for each cohort and each sample day or time combination, and the maximum change over the entire study was determined.

## Results

### Baseline and patient characteristics

**Fig. 1:**
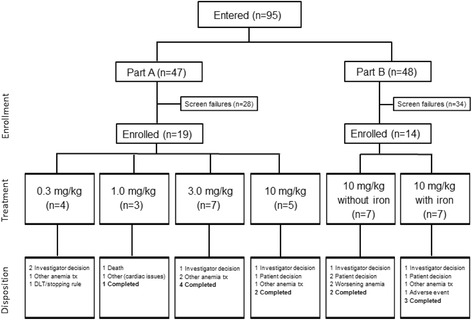
Study flow diagram

Between 19 January 2010 and 10 December 2014, a total of 33 patients were enrolled: 19 patients in part A and 14 patients in part B (7 patients each in cohorts B1 and B2) (Fig. 1). Table 1 summarizes their baseline and disease characteristics. Patients received a median of 4.5 prior oncology treatments. Overall, mean (SD) hemoglobin and erythropoietin levels at baseline were 9.2 (0.95) g/dL and 81.8 (88.29) mIU/mL, respectively.

**Table 1:** Baseline and disease characteristics by dosing group

	Part A				Part B		Total (*n* = 33)
	0.3 mg/kg (*n* = 4)	1 mg/kg (*n* = 3)	3 mg/kg (*n* = 7)	10 mg/kg (*n* = 5)	10 mg/kg without oral iron (cohort B1) (*n* = 7)	10 mg/kg with oral iron (cohort B2) (*n* = 7)	
Age, years	64.5 (58–75)	76.7 (63–88)	62.1 (44–78)	63.2 (53–75)	66.3 (50–79)	62.7 (51–72)	64.9 (44–88)
Sex							
Men	2 (50)	2 (67)	3 (43)	2 (40)	3 (43)	1 (14)	13 (39)
Women	2 (50)	1 (33)	4 (57)	3 (60)	4 (57)	6 (86)	20 (61)
Race							
White	4 (100)	3 (100)	5 (71)	3 (60)	5 (71)	5 (71)	25 (76)
Black or African American	0	0	2 (29)	1 (20)	2 (29)	2 (29)	7 (21)
Asian	0	0	0	1 (20)	0	0	1 (3)
ECOG performance status							
0	1 (25)	1 (33)	2 (29)	0	0	0	4 (12)
1	3 (75)	2 (67)	3 (43)	5 (100)	7 (100)	6 (86)	26 (79)
2	0	0	2 (29)	0	0	1 (14)	3 (9)
Prior cancer treatment							
Systemic chemotherapy	4 (100)	3 (100)	7 (100)	5 (100)	5 (71)	4 (57)	28 (85)
Radiotherapy	1 (25)	1 (33)	2 (29)	3 (60)	2 (29)	5 (71)	14 (42)
Surgery	3 (75)	1 (33)	2 (29)	2 (40)	1 (14)	2 (29)	11 (33)
Median no. of prior oncology treatments	9.5	4.0	6.0	5.0	2.0	1.5	4.5
Mean serum hepcidin (ng/mL)	24.9 (18.51)	12.2 (8.48)	36.4 (21.87)	51.1 (39.81)	45.4 (20.23)	39.7 (38.99)	37.6 (28.46)
Mean hemoglobin (g/dL)	9.1 (1.45)	8.9 (0.91)	8.5 (0.83)	9.8 (0.84)	9.4 (1.13)	9.5 (0.31)	9.2 (0.95)
Mean erythropoietin (mIU/mL)	141.3 (92.71)	42.0 (20.16)	103.2 (135.14)	94.4 (116.49)	70.0 (60.87)	46.1 (25.67)	81.8 (88.29)
Mean serum ferritin (μg/L)	779.0 (303.64)	237.2 (136.68)	667.1 (683.65)	263.7 (178.90)	714.6 (553.58)	289.0 (175.70)	510.3 (464.15)
Cancer type							
Multiple myeloma	1 (25)	0	2 (29)	3 (60)	3 (43)	3 (43)	14 (42)
Breast cancer	0	0	0	1 (20)	3 (43)	0	4 (12)
Other^a^	3 (75)	3 (100)	5 (71)	1 (20)	1 (14)	4 (57)	15 (46)

Values shown are for the safety population (i.e., all patients who received at least one dose of LY2787106 [*n* = 33]). Data are mean (range) for age; median for number of prior oncology treatments; mean (SD) for serum hepcidin, hemoglobin, erythropoietin, and serum ferritin; and *n* (%) for all other parameters

*ECOG* Eastern Cooperative Oncology Group

^a^“Other” cancer types include Waldenstrom’s macroglobulinemia (2 patients), pancreatic adenocarcinoma (2 patients), rectal cancer (2 patients), renal cell carcinoma, metastatic prostate cancer, colon adenocarcinoma, unknown primary presumed ovarian adenocarcinoma, metastatic sarcomatoid carcinoma of the distal esophagus, non-small cell lung cancer, adenocarcinoma of lung, gastrointestinal stromal tumor, and adenocarcinoma of the liver

### Safety

No DLTs were reported in part A, so the MTD was not reached. One patient in cohort B1 with liver adenocarcinoma experienced a non-treatment-related adverse event (grade 3 anemia) that was declared a stopping rule by the investigator during cycle 13. One patient in cohort B2 with lung adenocarcinoma experienced a possibly treatment-related serious adverse event (grade 3 cardiac failure) and a non-treatment-related adverse event (grade 3 aspartate aminotransferase increase) during cycle 11, which together the investigator considered a DLT-equivalent toxicity. Both patients recovered from the events.

Overall, the median number of treatment cycles given and completed was 3. Across dosing cohorts, the median ranged in increasing order from 1 cycle in the 0.3 mg/kg Q3W cohort (part A) to 14 cycles in cohort B2 (part B, 10 mg/kg weekly without iron).

Table 2 shows the most common treatment-emergent adverse events overall. The majority of adverse events were grade 1 or 2. The most common grade 3 adverse event was fatigue (1 patient in the 1 mg/kg cohort, 2 patients in the 3 mg/kg cohort, 5 patients in cohort B1, and all 7 patients in cohort B2). Of note, grade 3 decreased hemoglobin unrelated to treatment was reported in 2 patients (both in cohort B1). Also reported were grade 3 aspartate aminotransferase elevation (1 patient in cohort B2) and grade 1 increase in blood creatinine (1 patient each in cohorts B1 and B2), none of which were considered related to treatment. Only one grade 4 adverse event was reported (dyspnea considered unrelated to treatment, in the 0.3 mg/kg cohort). Two patients were discontinued from treatment, including 1 patient in cohort B2 with possibly treatment-related grade 3 cardiac failure (left ventricular systolic dysfunction, per NCI CTCAE terminology) and 1 patient in the 1 mg/kg cohort who died of disease progression. Treatment-related adverse events were reported in 4 patients (12.1%), including QT prolongation (grade 1) and neutropenia (grade 2) in 1 patient each in cohort B1 and increased creatine phosphokinase (grade 2) and cardiac failure (grade 3) in 1 patient each in cohort B2.

**Table 2:** Treatment-emergent adverse events regardless of causality reported by ≥10% of patients on LY2787106 (safety population)

Event	Part A				Part B		Total (*n* = 33)
	0.3 mg/kg (*n* = 4)	1 mg/kg (*n* = 3)	3 mg/kg (*n* = 7)	10 mg/kg (*n* = 5)	10 mg/kg without oral iron (cohort B1) (*n* = 7)	10 mg/kg with oral iron (cohort B2) (*n* = 7)	
Fatigue	0	1 (33.3)	2 (28.6)	0	5 (71.4)	7 (100)	15 (45.5)
Nausea	1 (25.0)	1 (33.3)	2 (28.6)	1 (20.0)	3 (42.9)	4 (57.1)	12 (36.4)
Dyspnea	1 (25.0)	0	0	0	4 (57.1)	5 (71.4)	10 (30.3)
Constipation	1 (25.0)	2 (66.7)	0	1 (20.0)	2 (28.6)	1 (14.3)	7 (21.2)
Edema	0	1 (33.3)	0	0	3 (42.9)	2 (28.6)	7 (21.2)
Abdominal pain	1 (25.0)	2 (66.7)	2 (28.6)	0	0	1 (14.3)	6 (18.2)
Headache	0	0	2 (28.6)	0	1 (14.3)	3 (42.9)	6 (18.2)
Hyponatremia	1 (25.)	0	1 (14.3)	0	2 (28.6)	2 (28.6)	6 (18.2)
Arthralgia	0	0	1 (14.3)	0	2 (28.6)	2 (28.6)	5 (15.2)
Bone pain	0	0	0	0	2 (28.6)	3 (42.9)	5 (15.2)
Diarrhea	0	0	0	0	2 (28.6)	3 (42.9)	5 (15.2)
Vomiting	0	0	0	1 (20.0)	2 (28.6)	2 (28.6)	5 (15.2)
Chills	0	0	0	0	1 (14.3)	3 (42.9)	4 (12.1)
Cough	0	0	1 (14.3)	0	2 (28.6)	1 (14.3)	4 (12.1)
Decreased appetite	2 (50.0)	0	0	0	2 (28.6)	0	4 (12.1)
Dizziness	0	0	2 (28.6)	0	1 (14.3)	1 (14.3)	4 (12.1)
Muscle spasms	1 (25.0)	0	1 (14.3)	0	0	2 (28.6)	4 (12.1)
Myalgia	0	0	1 (14.3)	0	1 (14.3)	2 (28.6)	4 (12.1)
Nasal congestion	0	1 (33.3)	1 (14.3)	0	1 (14.3)	1 (14.3)	4 (12.1)
Pain in extremity	1 (25.0)	0	0	0	0	3 (42.9)	4 (12.1)

Values shown are for the safety population (i.e., all patients who received at least one dose of LY2787106 [*n* = 33]). Data are *n* (%). Events are described using preferred terms per MedDRA version 17.1. Patients reporting more than one adverse event were counted only once

Serious adverse events were reported in 6 patients (18.2%), including 1 patient in the 0.3 mg/kg cohort (dyspnea), 2 patients in the 1.0 mg/kg cohort (chondrocalcinosis pyrophosphate [i.e., “exacerbation of pseudogout”] and neoplasm progression in 1 each), 2 patients in the 3 mg/kg cohort (pneumonia and arthralgia/myalgia in 1 each), and the 1 patient in cohort B2 who experienced cardiac failure. Of these serious adverse events, only the cardiac failure was considered possibly related to treatment by the investigator. However, even though the relatedness of the cardiac failure to LY2787106 could not be ruled out completely, both investigator and sponsor considered the event more likely due to pressure on the pulmonary artery from the patient’s preexisting lung tumor. Two deaths were reported: one in the 1 mg/kg cohort due to disease progression, as noted above, and one in the 3 mg/kg cohort due to an undetermined cause.

Eight patients generated anti-drug antibodies (ADAs) to LY2787106, including 2 patients treated at 0.3 mg/kg, 1 patient at 1 mg/kg, 2 patients at 3 mg/kg, and 3 patients at 10 mg/kg (1 patient each in part A and in cohorts B1 and B2). No patients had hypersensitivity reactions. No correlation was observed between the dose and the proportion of ADA-positive patients, and there was no evidence that ADA positivity was linked to lower LY2787106 exposure.

### Efficacy

**Fig. 2:**
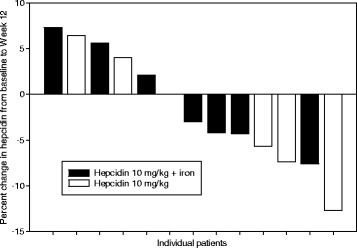
Percent change in hemoglobin level from baseline to end of cycle 4 (week 12). Values shown are for evaluable patients in study part B who received 10 mg/kg LY2787106 weekly (QW) without and with oral iron (cohorts B1 [*n* = 6] and B2 [*n* = 7], respectively). Only patients with a baseline and postbaseline result are included; for patients who did not reach week 12, their final assessment was used

As shown in Table 3, the mean change in hemoglobin level from baseline to the end of cycle 4 in part B was −0.5 g/dL (60% CI, −0.69, 0.31) without oral iron (cohort B1) and −0.2 g/dL (60% CI, −0.53, 0.07) with oral iron (65–72 mg of elemental iron daily, cohort B2). A ≥0.5 g/dL increase in hemoglobin levels was seen in 4 patients at week 12, including 2 patients in cohort B1 (each with multiple myeloma) and 2 patients in cohort B2 (1 with multiple myeloma and 1 with rectal cancer). In each of these cases, the change in hemoglobin levels was not sustained and reverted to baseline. No consistent increase in reticulocyte counts or changes in iron profiles were observed in these patients. Figure 2 shows the percent change in hemoglobin level from baseline to week 12 for all evaluable patients.

**Table 3:** Mean change in hemoglobin level from baseline to end of cycle 4

Hemoglobin (g/dL)	10 mg/kg without oral iron (cohort B1) (*n* = 6)	10 mg/kg with oral iron (cohort B2) (*n* = 7)	Total (*n* = 13)
Baseline^a^			
Mean (SD)	9.8 (0.71)	9.5 (0.31)	9.6 (0.53)
Range	8.8, 10.8	9.0, 9.9	8.8, 10.8
Change from baseline to end of cycle 4			
Mean (SD)	−0.5 (0.49)	−0.2 (0.80)	−0.4 (0.65)
60% CI for mean^b^	−0.69, −0.31	−0.53, 0.07	−0.53, −0.20

Values shown are for the evaluable population (i.e., patients in study part B who received all four of the first four per-cycle doses of LY2787106)

*CI* confidence interval, *SD* standard deviation

^a^Baseline is defined as the last reported measure before first dose

^b^Confidence intervals are based on the *t* distribution. Each side of a 2-sided 60% CI is the same as a 1-sided 80% confidence limit

### Pharmacokinetics

**Fig. 3:**
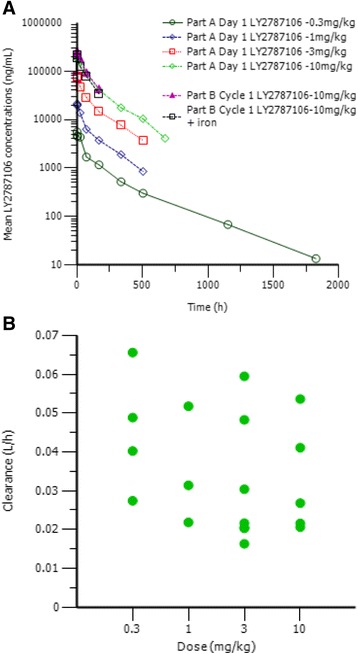
LY2787106 concentration-versus-time profile (**a**) and clearance-versus-dose scatter plot (**b**)

Pharmacokinetic parameters are summarized in Table 4. The PK profile of LY2787106 was consistent with reported PK characteristics of other mAbs; the profile in part A included a small clearance of 32 mL/h associated with limited volume of distribution, resulting in a t_1/2_ of 166 h (~7 days). As shown in Fig. 3, LY2787106 pharmacokinetics were independent of time and dose and exposure linear in the 0.3–10 mg/kg dosing range. On the basis of the mean t_1/2_ of 166 h, we calculated that mean accumulation ratios under weekly and Q3W dosing would be 1.98 and 1.14, respectively; the observed accumulation ratios of 1.55 (21%) and 1.2 (14%) (reported as mean [percentage coefficient of variation (CV%)]) for part B (weekly dosing) and part A (Q3W dosing), respectively, compared favorably with those estimates (Table 4).

**Table 4:** Pharmacokinetic parameters for LY2787106 after single dose and multiple doses

		Single dose						Multiple dose
Study part/dose	N	C_max_ (ng/mL)	AUC_0-inf_ (μg·h/mL)	AUC_τ,sd_ (μg·h/mL)	CL (mL/h)	t_1/2_ (h)	V (L)	AUC_τ,ss__(_μg·h/mL)
Part A								
0.3 mg/kg Q3W	4	5450 (24%) [4150–7160]	663 (42%) [414–1063]	597 (38%)	43 (38%) [28–67]	142 (55%) [75–260]	8.9 (23%) [6.8–11.7]	NA
1 mg/kg Q3W	3	22,300 (27%) [14,300–35,000]	2146 (64%) [799–5764]	1799 (50%)	33 (45%) [16–68]	213 (50%) [126–241]	10 (7%) [8.9–11.4]	2337 (41%) [1206–4530]
3 mg/kg Q3W	7	79,000 (15%) [105,000–71,000]	8934 (39%) [6772–11,788]	7991 (25%)	28 (52%) [19–40]	172 (54%) [71–375]	6.9 (21%) [5.9–8]	9755 (30%) [7402–12,856]
10 mg/kg Q3W	5	205,000 (12%) [183,000–231,000]	24,503 (19%) [20,485–29,309]	22,457 (16%)	30 (44%) [20–45]	154 (21%) [124–197]	6.8 (51%) [4.3–11]	26,560 (29%) [20,182–34,952]
All doses	19				32 (46%) [27–38]	166 (45%) [72–375]	7.7 (32%) [6.8–8.7]	
Part B								
10 mg/kg QW	6	258,000 (15%) [228,000–292,000]	21,000 (19%) [17,935–24,590]	16,723 (18%)	35 (29%) [27–44]	72 (18%) [57–87]	3.6 (34%) [2.7–4.7]	24,054 (21%) [19,754–29,289]
10 mg/kg QW + iron	5	199,000 (12%) [178,000–223,000]	16,798 (27%) [13,031–21,656]	13,261 (24%)	44 (22%) [36–55]	75 (15%) [68–96]	4.8 (23%) [3.9–6]	20,278 (51%) [11,550–35,600]
Accumulation ratios								
AUC_τ,ss_/AUC_τ,sd_								
Part A (Q3W)	13	1.2 (14%) [1.12–1.29]						
Part B (QW)	9	1.55 (21%) [1.36–1.76]						
AUC_τ,ss_/AUC_0-inf_	13	1.04 (15%) [0.97–1.12]						

Values for pharmacokinetic parameters are reported as geometric mean (CV%) [90% CI], except for t_1/2_ reported as geometric mean (CV%) [range]. Values for accumulation ratios are reported as mean (CV%) [90% CI]

*AUC*_*0-inf*_ area under the plasma concentration versus time curve to infinity after single dose, *AUC*_*τ,sd*_ area under the plasma concentration versus time curve over the dosing interval after single dose, *AUC*_*τ,ss*_ area under the plasma concentration versus time curve during one dosing interval at steady state, *CI* confidence interval, *CL* total body clearance, *C*_*max*_ maximum observed concentration, *CV%* percentage coefficient of variation, *Q3W* every 3 weeks, *QW* weekly, *t*_*1/2*_ mean elimination half-life associated with the terminal rate constant (λ_z_), *V* volume of distribution

### Pharmacodynamics

**Fig. 4:**
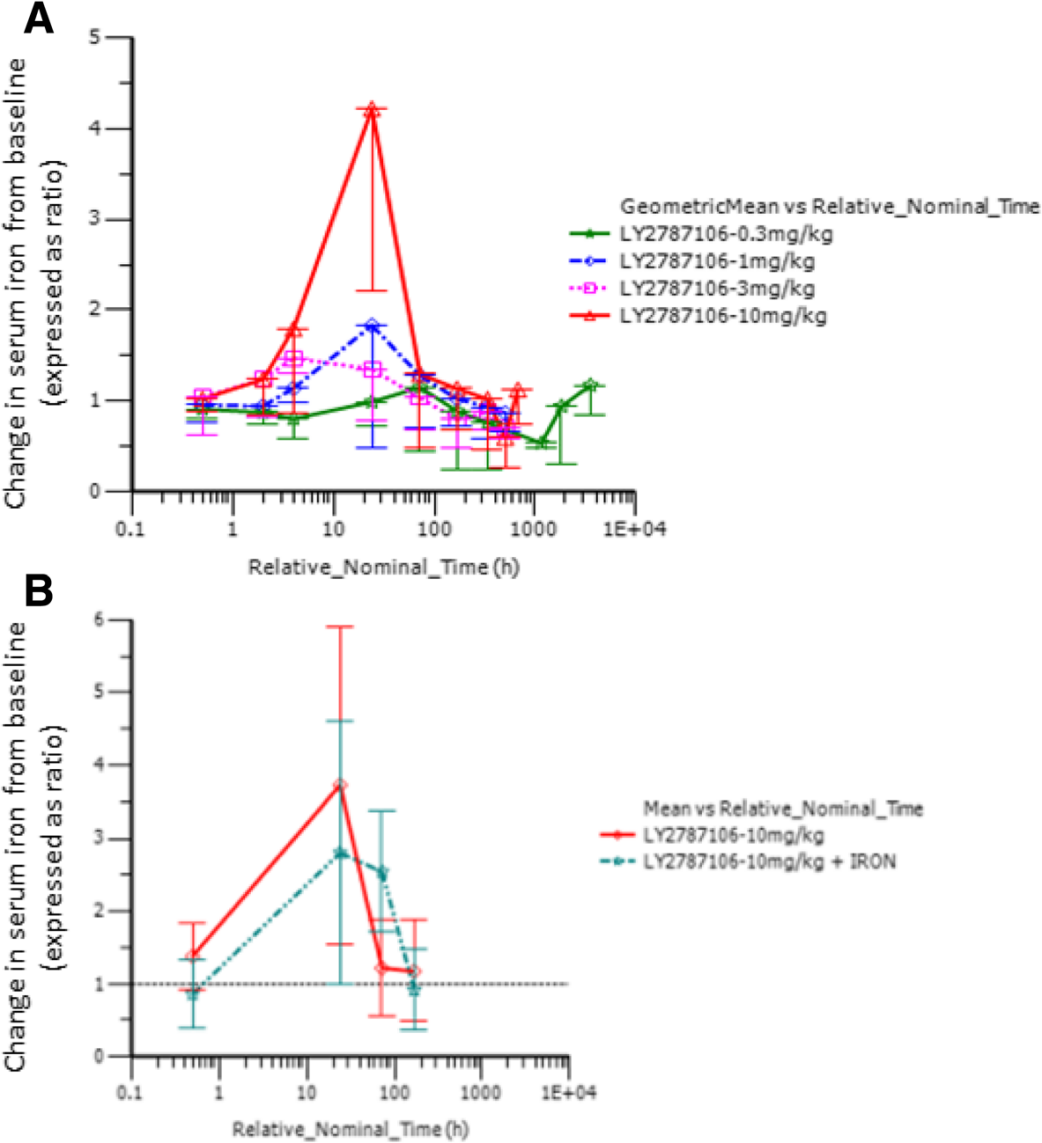
Change in serum iron level (expressed as ratio relative to baseline) versus time course following (**a**) a single dose and (**b**) multiple weekly doses (after 5th dose). Relative_Nominal_Time (h) is the protocol-scheduled time after last dose in hours presented on a log scale for greater clarity and better presentation of the earlier time point, where 168 h = 7 days = 1 week and 336 h = 14 days = 2 weeks. Data are displayed as mean ± SD

**Fig. 5:**
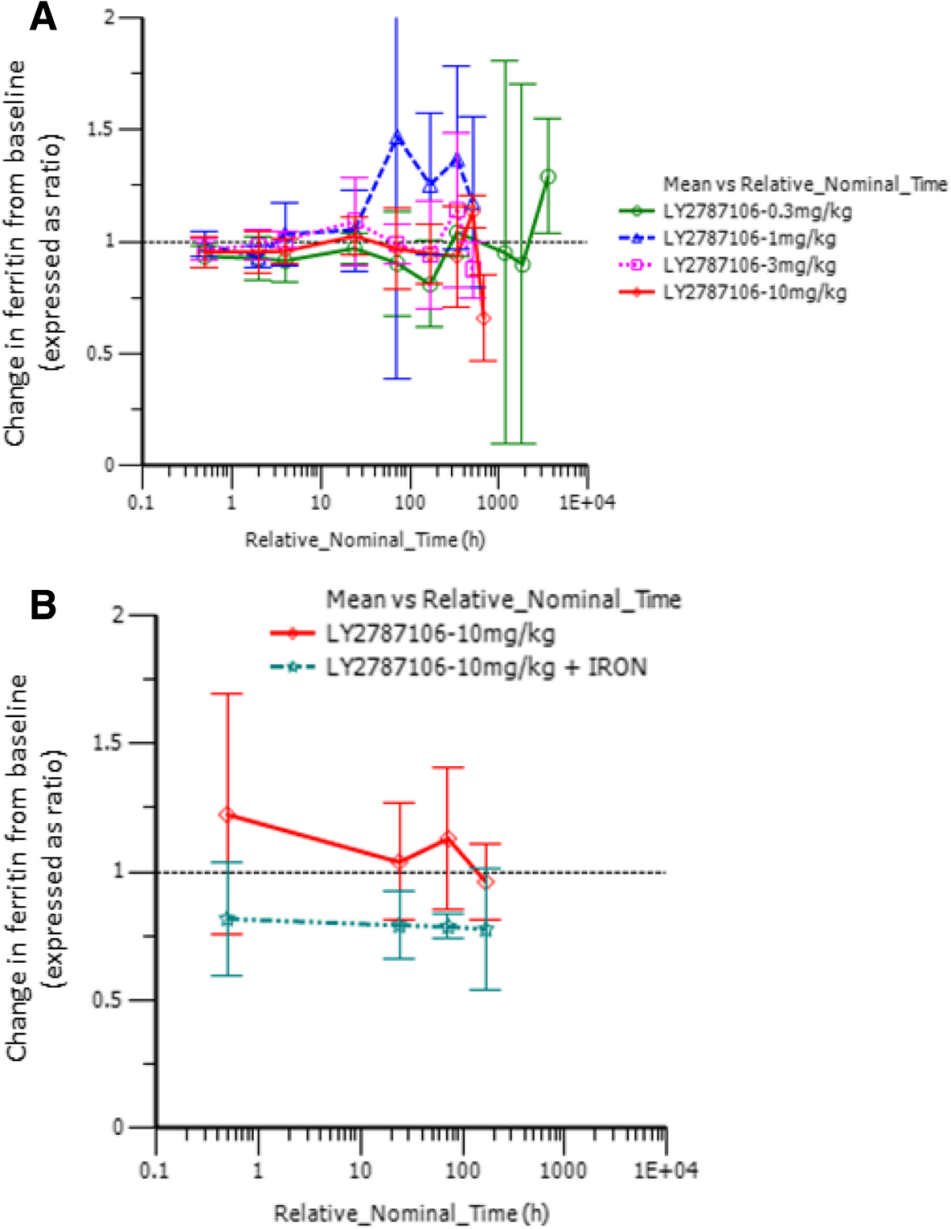
Change in ferritin level (expressed as ratio relative to baseline) versus time course following (**a**) a single dose and (**b**) multiple weekly doses (after 5th dose). Relative_Nominal_Time (hr) is the protocol-scheduled time after last dose in hours presented on a log scale for greater clarity and better presentation of the earlier time point, where 168 h = 7 days = 1 week and 336 h = 14 days = 2 weeks. Data are displayed as mean ± SD

**Fig. 6:**
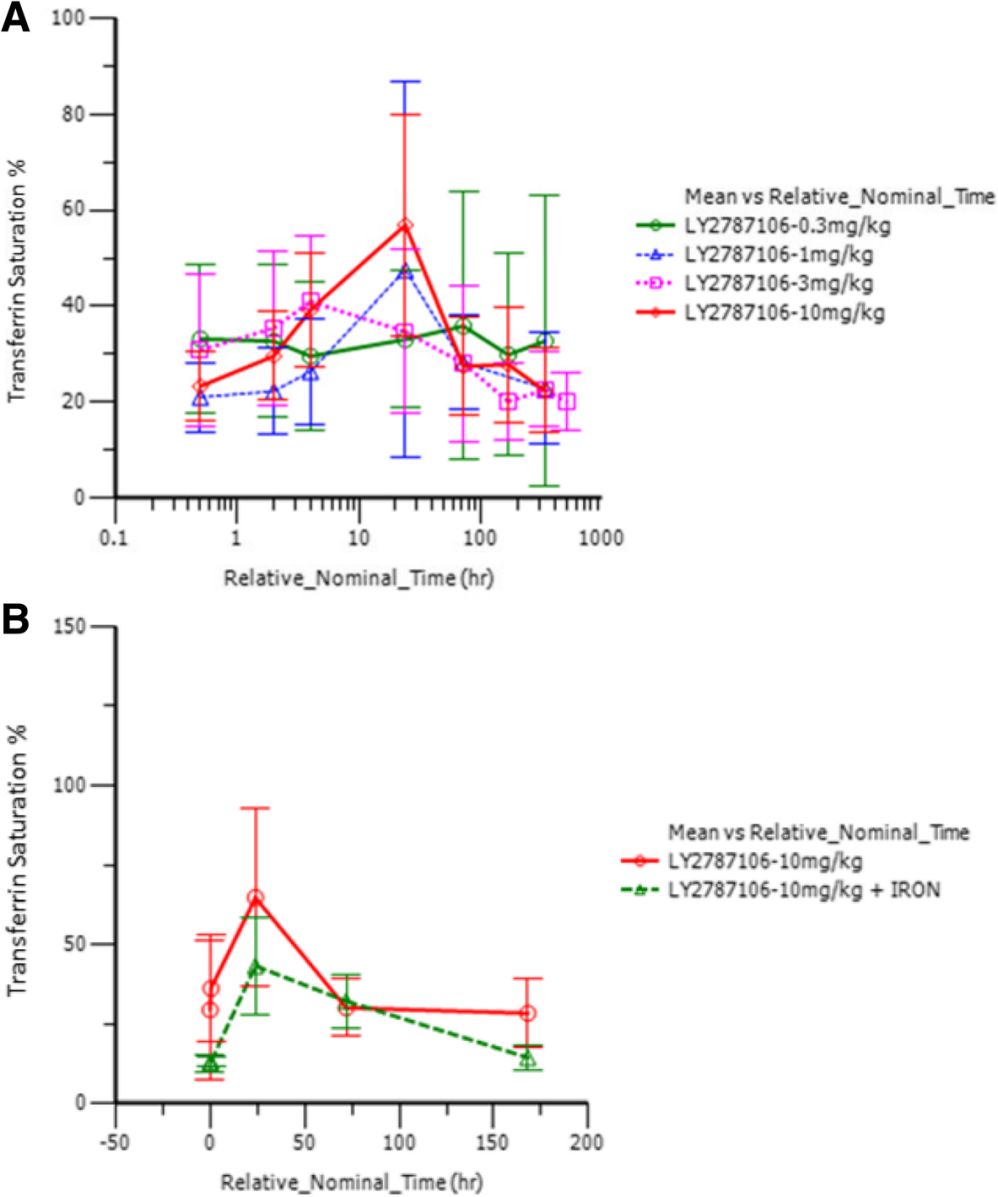
Transferrin saturation (percentage) concentration-versus-time profile following (**a**) a single dose and (**b**) multiple weekly doses (after 5th dose). Relative_Nominal_Time (hr) is the protocol-scheduled time after last dose in hours presented on a log scale for greater clarity and better presentation of the earlier time point, where 168 h = 7 days = 1 week and 336 h = 14 days = 2 weeks. Data are displayed as mean ± SD

**Fig. 7:**
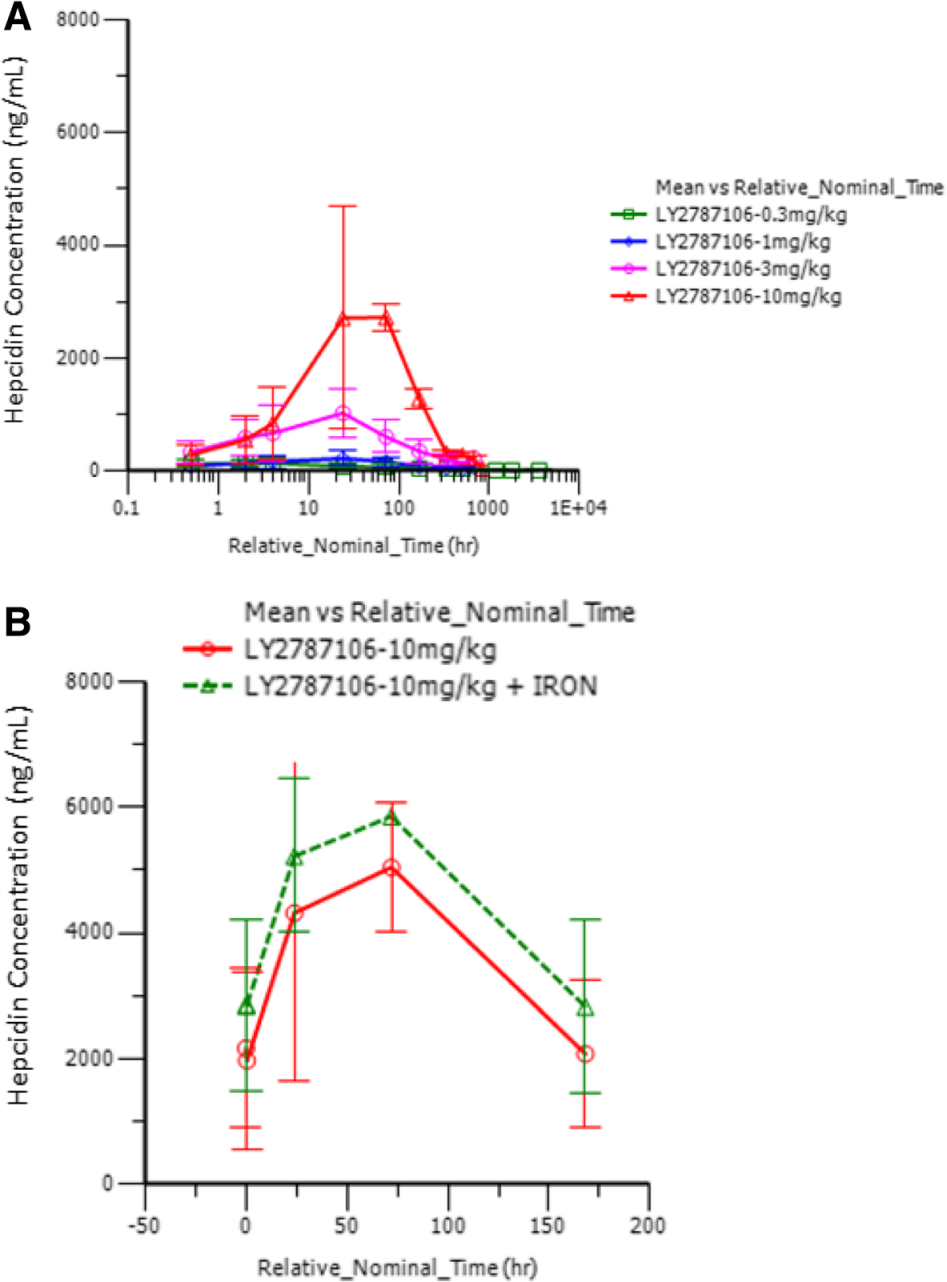
Hepcidin (ng/mL) concentration-versus-time profile following (**a**) a single dose and (**b**) multiple weekly doses (after 5th dose). Relative_Nominal_Time (hr) is the protocol-scheduled time after last dose in hours presented on a log scale for greater clarity and better presentation of the earlier time point, where 168 h = 7 days = 1 week and 336 h = 14 days = 2 weeks. Data are displayed as mean ± SD

Pharmacodynamic data indicated that LY2787106 administration at the highest dose (10 mg/kg) in parts A and B led to a mean maximum 3.48-fold increase (90% CI, 2.5–4.8) in serum iron from baseline (Fig. 4). The increase in serum iron peaked at approximately 24–48 h postdose, but returned to baseline within a week after dosing. Although the increase in serum iron was not accompanied by any changes in ferritin levels indicative of increased iron mobilization or storage (Fig. 5), it did parallel a transient increase in TSAT particularly at the highest dose of 10 mg/kg (Fig. 6). Generally, the reticulocyte count profiles after LY2787106 administration, represented as ratio relative to baseline, did not indicate a consistent increase in reticulocytes. However, a transient increase in the mean ratio of reticulocyte count relative to baseline was observed at the 2-week postdose time point in patients who received 10 mg/kg Q3W, but relatively little change in hemoglobin levels. In addition, an approximately 1.5- to 2-fold increase in reticulocyte count was observed at weeks 5 to 6 in patients given the 10 mg/kg weekly dose in cohort B1. However, in part B, none of the 4 patients who exhibited a ≥0.5 g/dL increase (albeit transient) in hemoglobin from baseline showed any consistent increase in reticulocyte counts. As expected, LY2787106 administration led to increased hepcidin concentrations secondary to hepcidin binding/neutralization by LY2787106 and hepcidin release in response to iron increase (Fig. 7).

Other biomarkers including IL-6, TNF-α, C-reactive protein, and erythropoietin showed no consistent trends in their levels relative to changes in hemoglobin, hepcidin, reticulocyte, and iron profiles.

## Discussion

This is the first clinical trial of a fully humanized mAb against hepcidin in cancer patients with anemia. In light of hepcidin’s importance in the pathogenesis of anemia, especially in cancer, we explored the feasibility of using a mAb to target hepcidin and assess its effect on iron and anemia. Using nonclinical data, a PK/PD model for LY2787106 was developed to determine the starting dose for this study. The model predicted minimal to no effect at 1 mg/kg and maximal effect between 10 and 30 mg/kg. Thus, four dose levels ranging from 0.3 to 10 mg/kg administered Q3W were selected for part A of the study.

Patients treated in the four dosing cohorts during the dose-escalation phase (part A) of this study tolerated the treatment well, as evidenced by the absence of DLTs and grade ≥3 treatment-related adverse events. Interim data from the study demonstrated a significant increase in serum iron levels at the 10 mg/kg dose level within 24 h after LY2787106 infusion (see Additional file 1: Figure S1). However, the increase in serum iron levels was transient and returned to baseline in less than 8 days. These findings coincided with inconsistent response in reticulocyte count and hemoglobin levels in the patients treated in part A.

In part B of the study, only approximately half of the planned 24 patients were enrolled, mainly because of slow accrual. The safety and tolerability of LY2787106 observed in part A were further confirmed by the relative infrequency of treatment-emergent adverse events, which were primarily grade 1/2 in severity. Serum iron and TSAT increased a few hours after administration of LY2787106, which confirmed that the administration of 10 mg/kg LY2787106 adequately neutralized hepcidin. However, these changes remained transient and did not affect ferritin levels, hemoglobin values, or reticulocyte counts in either dosing group despite the increase to once weekly dosing and the addition of supplemental oral iron in cohort B2. Similar observations regarding serum iron and TSAT were made in a study of an anti-hepcidin pegylated structured mirror-image oligoribonucleotide, lexaptepid pegol, in healthy patients [10]. In that study, however, an increase in serum ferritin levels from baseline was seen with lexaptepid pegol that was not seen in the present study with LY2787106. This difference may be due to elevated baseline serum ferritin levels typically observed in cancer patients and other confounding factors such as concomitant chemotherapy [11]. In addition, the lack of sustained mobilization of serum iron may have contributed to the lack of changes in serum ferritin levels.

Several factors may have contributed to the lack of hematologic response in this study: the unsustained increase in serum iron levels despite weekly dosing, myelosuppression due to concomitant chemotherapy, the relative frequency of phlebotomies on study, the presence of comorbidities that could have interfered with erythropoiesis, and the heterogeneity of cancer types with distinct inflammatory profiles [12]. Circadian changes may also have influenced the complex regulation between serum iron and hepcidin levels. Other studies have shown elevated levels of cytokines such as IL-6 in cancer patients with anemia [13], which may contribute to a lack of significant response in erythropoiesis and to an increase in hepcidin expression levels [14]. Therefore, neutralizing hepcidin alone may not be sufficient to induce robust and sustained elevation of hemoglobin and reticulocytes in patients with cancer. Furthermore, compensatory increase in the hepcidin levels may also explain the lack of significant response in hemoglobin levels. The incomplete enrolment in part B due to slow accrual may have limited the number of patients evaluable for hematological changes. However, its impact on the overall findings is minimal compared with the effects of other confounding factors as mentioned previously.

The lack of a consistent reticulocyte and hemoglobin response in this study also led us to investigate whether the dose we used was appropriate. We did this by examining the target inhibition in terms of hepcidin/LY2787106 ratios in two possible scenarios. In the first scenario, which assumed that each drug molecule bound only one hepcidin molecule, we observed mean (90% CI) hepcidin/LY2787106 ratios of 0.5 (0.23–1.48), 1.6 (1.2–2.2), and 1.3 (0.83–1.73) at 24, 72, and 168 h, respectively, postdose. In the second scenario, which assumed that each drug molecule bound two hepcidin molecules, we observed mean ratios of 0.25 (0.115–0.74), 0.8 (0.6–1.1), and 0.65 (0.415–0.865) at 24, 72, and 168 h, respectively. Since most of the CIs around these mean ratio values were below or included 1, we postulate that the 10 mg/kg dose of LY2787106 was likely sufficient to significantly neutralize hepcidin. This hypothesis is true under the assumption, albeit unverified, that the hepcidin/LY2787106 ratios in serum are an adequate surrogate to the ratio at the level of the site of action of hepcidin (i.e., the level of the ferroportin receptor). The significant increase in iron and TSAT levels at the 10 mg/kg dose of LY2787106 further supports that hypothesis. One strategy for testing the hypothesis would have been to escalate the LY2787106 dose beyond 10 mg/kg to determine whether or not the pharmacological response was optimal/maximal at 10 mg/kg. However, we did not do so because we expected to see an improvement in PD response with weekly (as opposed to Q3W) dosing if the optimal PD effect had not been reached at 10 mg/kg. Indeed, despite the significantly higher accumulation of LY2787106 under weekly versus Q3W dosing (see Table 4), the changes in iron levels were very similar after LY2787106 administration for both dosing intervals (weekly and Q3W).

## Conclusions

In conclusion, LY2787106 was well tolerated in cancer patients with anemia. Targeting the hepcidin-ferroportin pathway by neutralizing hepcidin resulted in transient iron mobilization and reticulocyte count relative to baseline, thus supporting the role of hepcidin in iron regulation. Agents that affect other relevant targets in the hepcidin-ferroportin pathway such as ferroportin are currently being evaluated.

## Additional file

Additional file 1: Inclusion Criteria. Exclusion Criteria. Stopping Rules. **Figure S1.** Changes in (A) serum iron and (B) transferrin saturation from baseline in 3- and 10-mg/kg dosing cohorts in part A, as determined at interim analysis before start of part B. (DOCX 336 kb)

## Abbreviations

ADA: Anti-drug antibody
AUC: Area under the concentration-time curve
CHr: Reticulocyte hemoglobin content
CI: Confidence interval
CL: Clearance
C_max_: Maximum concentration
CV%: Percentage coefficient of variation
DLT: Dose-limiting toxicity
ECOG: Eastern Cooperative Oncology Group
IL-6: Interleukin-6
IV: Intravenously
MedDRA: Medical Dictionary for Regulatory Activities
MTD: Maximum tolerated dose
NCI CTCAE: National Cancer Institute Common Terminology Criteria for Adverse Events
PD: Pharmacodynamic(s)
PK: Pharmacokinetic(s)
Q3W: Every 3 weeks
QW: Weekly
RBC: Red blood cell
t_1/2_: Half-life
TIBC: Total iron binding capacity
TNF-α: Tumor necrosis factor-α
TSAT: Transferrin saturation
V_d_: Volume of distribution
