# Supplementary material for: A first-in-human phase 1 study of a hepcidin monoclonal antibody, LY2787106, in cancer-associated anemia
Source: J Hematol Oncol. 2017 Mar 21;10:73. doi: 10.1186/s13045-017-0427-x (PMC5361694; doi:10.1186/s13045-017-0427-x)
Supplement: Additional file 1: — Inclusion Criteria. Exclusion Criteria. Stopping Rules. Figure S1. Changes in (A) serum iron and (B) transferrin saturation from baseline in 3- and 10-mg/kg dosing cohorts in part A, as determined at interim analysis before start of part B. (DOCX 336 kb) [file 13045_2017_427_MOESM1_ESM.docx]

# **Additional file 1**

## Inclusion Criteria

Patients were eligible to be included in the study only if they met **all** of the following criteria within 28 days of study enrollment, or as otherwise defined by a specific criterion:

- Had histological or cytological diagnosis of non-myeloid cancer (solid tumors, lymphomas, or multiple myeloma) that was metastatic and/or incurable.
- Had previously been treated with at least 1 systemic (oral, IV, or SQ) anticancer therapy or regimen.
- Had hemoglobin <11 g/dL. Patients with hemoglobin <7.0 g/dL who refused or were not eligible for a red blood cell transfusion could be eligible after discussion with the sponsor if they met all other eligibility criteria prior to study entry.
- Had a hepcidin level ≥5 ng/mL.
- Were ≥18 years of age.
- Had given written informed consent prior to any study-specific procedures.
- Had adequate organ function including:
- Hematologic: Absolute neutrophil count (ANC) ≥1.5 × 10^9^/L and platelets ≥75 × 10^9^/L. Platelet transfusions were not allowed within 7 days of enrollment to reach 75 × 10^9^/L.
- Hepatic: Bilirubin ≤1.5 × upper limit of normal (ULN), alanine transaminase (ALT) and aspartate transaminase (AST) ≤2.5 × ULN.
- Renal: Serum creatinine ≤1.5 × ULN.
- Had serum erythropoietin, folate, B12, and ferritin levels ≥ lower limit of normal, and transferrin saturation >10% but ≤60%.
- Had an Eastern Cooperative Oncology Group (ECOG) performance status ≤2.
- Were reliable and willing to be available for the duration of the study and were willing to follow study procedures.
- If male or female with reproductive potential, had to agree to use medically approved contraception during the trial and for 4 months following the last dose of study drug.
- If female with child bearing potential, had a negative serum pregnancy test ≤7 days prior to the first dose of study drug.
- Had an estimated life expectancy of ≥12 weeks.

## Exclusion Criteria

Patients were excluded from the study if they met **any** of the following criteria within 28 days of study enrollment, or as otherwise defined by a specific criterion:

- Had received treatment in the previous 21 days with, or had not recovered fully from, a drug that had not received regulatory approval for any indication.
- Had received erythropoietin‑stimulating agents (ESAs) in the previous 21 days or red blood cell transfusions in the previous 14 days, or in the investigator’s opinion, were likely to need red blood cell transfusion more frequently than every 21 days.
- Had received parenteral iron supplementation within the 14 days prior to enrollment.
- Had a documented history of pure red cell aplasia, thalassemia major, or sickle cell disease.
- Had a history of cirrhosis or major organ transplantation.
- QTc >470 msec
- Had evidence of clinically significant hemolysis or bleeding.
- Had a clinically significant infection within 14 days of enrollment.
- Had a suspected or confirmed history of hemochromatosis.
- Had other serious preexisting medical conditions (left to the discretion of the investigator).
- Had symptomatic central nervous system malignancy or metastasis (screening not required).
- Had acute or chronic leukemia.
- Was a female who was pregnant or lactating.
- Had a history of human immunodeficiency virus (HIV), hepatitis B, or hepatitis C (screening not required).
- Had received external beam radiotherapy to more than 25% of the bone marrow.
- Had known clinically significant hypersensitivity to biologic agents.
- Had received live vaccine(s) within 1 month of screening or had plans of doing that during the participation in the study.
- Had a history of congestive heart failure with New York Heart Association (NYHA) Class >2 (NYHA Class 1 and 2 were eligible), unstable angina or recent myocardial infarction (within 1 year prior to administration of study drug).

## Stopping Rules

Stopping rules for individual patients were defined as follows:

- Hemoglobin level >12 g/dL on Day 1 of 2 consecutive cycles or any hemoglobin level ≥13.0 g/dL in the absence of volume depletion (dehydration), and predose transferrin saturation >90% on Day 1 of 2 consecutive cycles that was unrelated to anticancer therapy.
- A dose-limiting toxicity (DLT)-equivalent toxicity (defined as a toxicity that met the criteria for a DLT but occurred in Cycle 2 or beyond in Part A or at any time in Part B).

# Figure S1


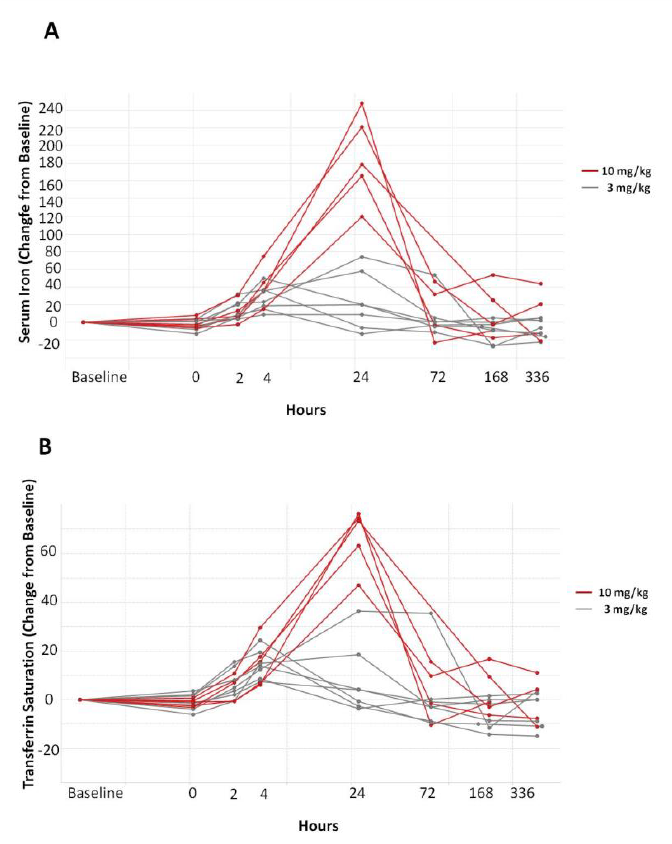


**Figure S1.** Changes in (A) serum iron and (B) transferrin saturation from baseline in 3- and 10‑mg/kg dosing cohorts in Part A, as determined at interim analysis before start of Part B (interim data cutoff date 29 March 2012). Consistent dose-dependent increases in serum iron and transferrin saturation were seen at the 3- and 10‑mg/kg dose levels, typically peaking within 24 hours after LY2787106 administration and returning to baseline by Day 8.
